# Medical Education

**Published:** 1897-08

**Authors:** 


### Medical Education.

Many persons are convinced that only a classic training in the university colleges can properly fit a man for the study of medicine. Now we are told by the deans of some of these colleges that their system of training is very imperfect and crude in its methods; also that they are anxiously looking around for improvements and trying to break away from the prestige and old-time forms of teaching.

There is something very refreshing in this awakening of educators in the higher centers of learning. The dogmatic egotism which has centered around some of the great universities of this country is lifting and we begin to realize that educational method in its highest forms are still very crude. While medical training and the standards of medical colleges are very far from the ideals which have been marked out, there is a restless movement and struggle upward which promises much for the future.

The medical men who urge so strenuously that a classic training in a literary college is an absolute essential as a foundation for a medical training are very often good examples of the folly of such training. Men with literary degrees from the best colleges are very often poor, weak medical men. The prize and honor men of the great literary colleges are rarely heard of in after-life in any department of human activity. Literally, the high classic training demanded as a requisite to enter upon the study of medicine is often a most serious obstacle to all future success. The fault is in the college methods, which have literally unfitted the man for close, hard work and broad reasoning. They have cultivated a strong personal bias in his capacity and superiority that is fatal to good work afterward. Fortunately this does not occur in all cases. Many college graduates rise above the narrowness of so-called culture and make a culture and acquire an independent knowledge for themselves.

We do not wish to discourage a thorough preliminary training before entering upon the study of medicine; but the idea that a degree from any college is evidence of such training is not true in many cases. While the medical colleges are extending their courses and requiring more thorough preparation before entering, they can not reach the end sought for in thoroughly educated profession by accepting the diplomas of colleges without question or examination. While we all recognize that graduates of the leading medical colleges may be very scantily equipped for the practice of medicine, it is a notorious fact that men with diplomas from the most famous institutions in this country are often very weak and uncultured. This is true the world over—the English or German student who has spent ten or more years at the great seats of learning may not have acquired the first principles of culture.

The idea can not be repeated too often, that no training can make a medical man who has not some constitutional adaptability and tact for the work. No colleges can graduate educated, cultivated men by the present indiscriminate methods applied to all, without regard to ability or personality. What will be required in the future will be some test of the character and capacity of the student for the life-work he would engage in; then training becomes exact and development and culture positive and certain.—*Journal of the American Medical Association.*

